# Mapping Thyroid Changes in Size and Position During Enlargement in Adult Mice With Hyperthyroidism

**DOI:** 10.1210/endocr/bqae062

**Published:** 2024-05-24

**Authors:** Zhongmin Li, Clara Wenhart, Andreas Reimann, Silvia Goebel, Yi-Li Cho, Goetz Muench

**Affiliations:** Advancecor GmbH, 82152 Martinsried, Germany; Advancecor GmbH, 82152 Martinsried, Germany; Advancecor GmbH, 82152 Martinsried, Germany; Advancecor GmbH, 82152 Martinsried, Germany; Advancecor GmbH, 82152 Martinsried, Germany; Advancecor GmbH, 82152 Martinsried, Germany

**Keywords:** thyroid, hyperthyroidism, thyroid volume, mouse, thyroid size, anatomy

## Abstract

The thyroid in Graves’ disease undergoes a considerable divergence in size and position from the normal anatomy. However, knowledge of the pathological anatomy related to the change, which is required before planned surgical or local intervention, or diagnosis, is neglected. To investigate Graves’ disease, we established a model of mice that successfully mimicked all the signs presented in the clinic. Under a long-term immunization (35 weeks), the animals displayed large heterogeneity in thyroid size, such as the cases of natural occurrence. These thyroids in the model were sized into various phases and registered. A blend of the registered thyroids and the thyroid and tracheal cartilage landmarks led to the production of site-dependent incidence graphs of thyroid in the front view and on the section for each phase. The merger of the incidence graphs of all the phases resulted in thyroid phase-dependent topography. The depicted graphs illustrate the fine localization of the thyroid in various sizes and their dynamic changes during enlargement, which may facilitate currently used fine-needle aspiration biopsy and ultrasonography-guided biopsy techniques. Familiarity with this knowledge might avoid misclassifying an abnormality as normal, or vice versa, and be helpful for imaging diagnosis and local surgery therapy in Graves’ disease.

The mouse thyroid gland has 2 lateral lobes, normally lying under the sternohyoid and sternothyroid muscles and lateral to the trachea just below the thyroid cartilage (1). The thyroid gland in normal adult mice is approximately 2- to 3-mm long, with sizes ranging from 2 to 3 mm³ (2). However, enormous growth in the state of hyperthyroidism sometimes achieves a volume of more than 6 times larger than the normal status (2). Such enlargement of the thyroid gland, which may be grossly obvious, is called a goiter and is 1 of the most common manifestations of thyroid disease. The enlarged thyroid represents considerable variability in anatomical position. Knowledge of the substantial anatomic variation is fundamental for the disease's accurate diagnosis and treatment.

However, pathological anatomy on the thyroid location following the alteration of its size is conspicuously neglected in literature because the thyroid’s roles in Graves’ disease in the metabolism, growth, regulation of certain electrolytes, treatment, and involvement in many disease processes has been highly emphasized.

For investigation of hyperthyroidism, a long-term model for human Graves´ disease is successfully established in mice using 3- and 4-weekly immunizations with recombinant adenovirus expressing the extracellular A subunit of the human thyrotropin receptor (Ad-TSHR) (2). In response to injections of Ad-TSHR, generation of TSHR-binding stimulatory antibodies, elevated serum thyroxin levels, goiter, histological thyroid alterations, cardiac involvement, and Graves’ eye disease are observed (2-6). The features observed in the model simulate all the signs shown clinically.

To study the pathological anatomy of the thyroid location following the alteration in size, we adopted 4 strategies: the first was to prepare the thyroid cartilage skeleton and cricoid cartilage, which are the landmarks for orientation and localization of the thyroid; the second was to optimize an appropriate immunized period by comparing spectrums of thyroid size developed in different time points; the third was to size the thyroids by the optimized immunization into various phases and to produce the phase-dependent topography by digital combination of the registered thyroids and the cartilage landmarks; and the fourth was to validate the experiment protocol.

## Materials and Methods

### Animal Preparation

All animal experiments were performed in accordance with Directive 2010/63/EU and approved by the Government of Upper Bavaria in Munich, Germany (reference number: 55.2-1-54-2531-25-12), based on prior evaluation of animal study plan design and group sizes by a certified biostatistician. All protocols regarding animal handling and experiments were reported in accordance with ARRIVE guidelines (7).

Female BALB/c mice were purchased from Charles River, Sulzfeld, at the age of 5 weeks and acclimated for 1 week to start experiments at an age of 6 weeks. Animals were kept under standard housing conditions (23 ± 2 °C, 55 ± 10% rate of humidity) in groups of 10 animals each in GR1800DD cages (Tecniplast, UK).

**Figure 1.**
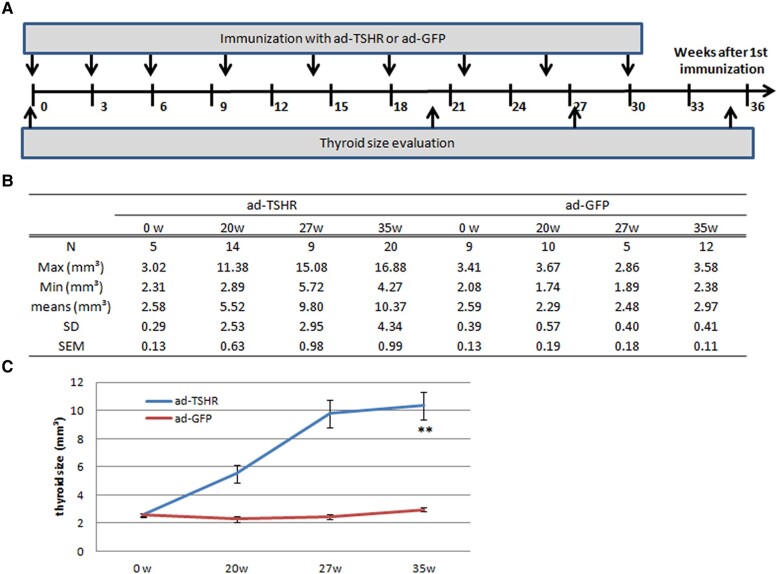
Schedule of immunization and thyroid size evaluation. (A) The time course of immunization and thyroid collection for volume measurement. (B) Profile of thyroid numbers and volumes (mm³) at each time point. (C) Thyroid volume alternation after immunization scheduled. **, vs 0 and 20 weeks, for all, *P* = .00022-.001, N = 5-20.

To determine the spectrum of thyroid volume in the period 0 to 35 weeks of immunization, mice were randomly distributed into 2 groups. One group of 48 mice received 10^10^ plaque-forming units of Ad-TSHR (Advancecor GmbH, Martinsried, Germany), and the other group of 36 mice that received 10^10^ plaque-forming units of Ad-GFP (Advancecor GmbH) as controls. The time course for the detailed immunization schedule was summarized in Fig 1A.

For the immunization, mice were anesthetized with isoflurane (introduction 5%, maintenance 1.5-2%) and placed on a heating pad. The adenovirus was injected into the left or right femoral muscles in a volume of 25 µL each time. In the entire experiments process, the animals were treated without other interventions except for blood withdrawal for serum analysis (reported separately elsewhere (2)) or injection of saline for mock controls. At the end of the time points piloted for thyroid size evaluation (Fig 1B), the animals were euthanized in deep anesthesia (170 mg/kg ketamine and 17 mg/kg xylazine).

To demarcate thyroids in various phases for determination of phase-dependent topography, in another separate experiment, 60 mice (Supplementary Table S1 (11), Supplementary Fig S1 (11)) were immunized with Ad-TSHR for 35 weeks in the style described in Fig 1A. In addition, 10 age-matched native mice served as controls.

For drawing of a cartilage skeleton and experiment validation, 6 female BALB/c mice and 6 male C57BL/6J mice were used, respectively. The background of these mice is summarized in Supplementary Table S2 (11).

### Dissection of Thyroid

A good thyroid gland definition is a prerequisite for successful localization. However, some thyroid glands are not clear and distinguishable in vivo on the borders from the front view. The reason for such a phenomenon may result from overlying tissue or the geometric configuration of the imaging system. Isolation of the thyroid is 1 solution for better observation.

Dissection of the thyroid glands was performed under a stereomicroscope after macroscopic observations had been completed. Before the dissection, the mouse neck was kept in a supine position with the chin straight up. Displacement of the animal's neck may lead to a wrong interpretation of the position of the gland. After clearing up the salivary glands and cutting away the connecting strap muscles (sternohyoid and sternothyroid muscles) running along the neck with scissors under the microscope, the trachea was exposed. To avoid the phenomenon that the trachea—an elastic tissue that might become shortened in the separated state ex vivo—we fixed the thyroid, trachea, and esophagus in situ by dropping 4% paraformaldehyde on the organ’s surface and incubating for 10 to 15 minutes. We dissociated the lateral muscles and gently grasped the trachea inferiorly with forceps. Crosscuts at the level above the larynx and the seventh cartilage ring were made, and the gland, along with the esophagus, pharynx, and trachea, was removed from the neck. The thyroid was left untouched and intact. Then the tissue block was put into 4% paraformaldehyde and incubated for 30 minutes before it was washed in PBS 3 times, photographed with a black background and the thyroid cartilage up, and embedded in OCT (Tissue-Tek Oct compound, Cat. No 4583, Sakura, IMEA).

### Drawings of a Thyroid Cartilage Skeleton and a Cricoid Cartilage Ring

**Figure 2.**
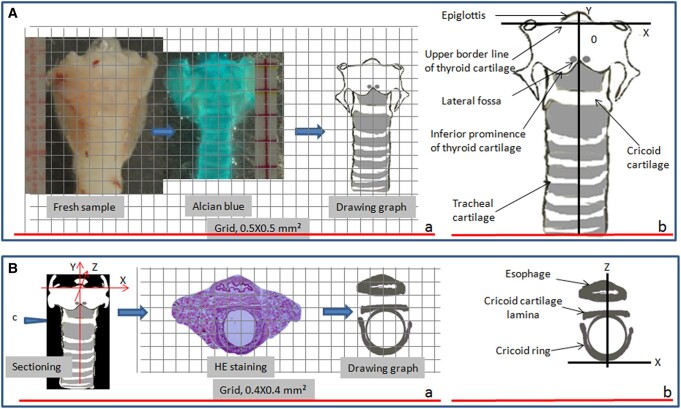
Protocol for sketching a cartilage skeleton and a cricoid cartilage ring and demonstration of the landmarks. (A) The age-matched specimens randomly collected were subjected to Alcian blue staining and a skeleton in front view was sketched using a coordinate or checked paper (Aa). For the orientation of the image blend, X and Y axes were drawn and overlain on the skeleton (Ab). The X and Y axes are the lines along the upper border and the anterior middle ridge of the thyroid cartilage, respectively. The lateral fossa is formed in the joints of the inferior prominence and anterior middle ridge of the thyroid cartilage. It is 0.9 mm in distance from the Y axis. (B) Similarly, the cricoid cartilage ring in the section was drawn (Ba). The section was collected in the cutting plane as “c” indicates. Under the setting of the space rectangular coordinates (X, Y, Z), illustrated in the left panel, Z and X axes were drawn and overlain on the ring for orientation (Bb). The X axis is the line parallel to the lamina and through the tangent point in the anterior wall of the cartilage. The Z axis is a line perpendicular to the cricoid cartilage lamina and through the tangent point.

To locate the thyroids, it is necessary to set the corresponding landmarks—the skeleton of the thyroid and tracheal cartilage. For drawings of the skeleton, we randomly selected 6 age-matched mice (Supplementary Table S2 (11)) and dissected the thyroid, along with the thyroid, cricoid, and tracheal cartilage. Following Alcian blue staining (described later), we found that the cartilage skeletons, which stained light blue, were quite similar among the stained samples (Supplementary Fig S2 (11)). A sketch of the skeleton was drawn at full size using an overlain coordinate (Fig 2Aa). For the landmark of sections through the cricoid cartilage ring, we took a counterpart section stained with hemoxylin and eosin for a prototype and sketched a cricoid cartilage ring in a similar way to the drawing of the skeleton mentioned previously (Fig 2Ba). The sections were collected at the cutting plane of the cricoid cartilage ring. The key landmarks were displayed in Fig 2Ab and 2Bb.

Under the setting of the space rectangular coordinate (left panel in Fig 2Ba), as similarly reported (8), X- and Y-axes were superposed on the skeleton. The X-axis is a parallel line to the supper border line of the thyroid cartilage, the Y-axis is the neck middle line (along the anterior middle line of the trachea), and the virtual initial point is the joint point of X- and Y-axes (Fig 2Ab). The Z-axis is a line perpendicular to the X and Y axes and through an original point, where the X and Y axes are joined (left panel in Fig 2Ba).

For the section that hits the cricoid ring, the X-axis is the line parallel to the lamina and through the tangent point in the anterior wall of the cartilage. The Z-axis is a line perpendicular to the cricoid cartilage lamina and through the tangent point (Fig 2Bb). They function for localization of the thyroid gland and orientation of the following image blend.

### Thyroid Embedding and Cutting

After washing, we placed the thyroid tissue block in OCT and incubated it for 1 hour under sonication to allow the chambers inside the trachea and esophagus to equilibrate with OCT. For the sonication, we put tubes, which contained the thyroid tissue blocks and OCT, into a water-filled sink of a sonicator (Bandelin Sonorex, Berlin, Germany). Using a 2-time embedding technique (8), the sample was embedded with an orientation of the larynx cartilage upper boundary down and the seventh trachea ring up (the long axis of the trachea is perpendicular to the cutting plane) on dry ice. The frozen specimen blocks were placed in a −80 °C freezer until ready to section.

Serially transverse sections were cut, starting from the upper boundary of larynx cartilage to the seventh tracheal cartilage ring with an interval of 500 µm as reported (2, 9), covering a distance of 6.5 mm. While cutting, we set the cutting temperature of the microtome (CM 1850 cryostat, Leica) to −19°C. If a complete and symmetric section was not visible, we repositioned the pedestal. Once a complete and symmetric cartilage section was visible, we set the cutting thickness to 5 µm and started collecting the sections. Sequential 5-µm sections were mounted on polylysine-coated slides (Cat. No J2800AMNZ, Thermo Scientific). Except for the first 10 serial sections collected, we discarded the following 90 serial sections (in the space of about 500 µm) and saved 10 serial sections again. Once the thyroid tissue disappeared on the slide, we stopped cutting. The slides were stored in a −80°C freezer until used for hematoxylin and eosin staining.

### HE Staining

We picked up the slides from the refrigerator and balanced them at room temperature for 30 minutes to dry. Tissue sections were dehydrated in absolute ethanol and rehydrated in distilled water each for 2 minutes, incubated in Harris´ hematoxylin solution (Cat. No HHS, Sigma-Aldrich) for 5 minutes, drained, and rinsed in distilled water. The tissue sections were then quickly dipped in 0.5% aqueous hydrochloric acid solution and washed in running tap water for 10 minutes. Finally, the sections were agitated in an eosin staining solution (Cat. No HT110-1, Sigma-Aldrich) for 20 seconds and drained for 5 seconds. Dehydration in increasing concentrations (95% and 100%) of alcohol was performed, followed by incubation in xylol for 2 minutes. We placed a drop of Permount (Cat. No 06522, Sigma-Aldrich) on the tissue section using a pipette and angled the coverslip to avoid air bubbles. The section was ready for observation and photographing after drying on air overnight.

### Observation and Photography

Hematoxylin and eosin-stained sections were examined using a bright field illumination on a Zeiss upright microscope. Fields of view covering the whole thyroid tissue in a section were selected and captured at lower magnification (×2.5) using the Zeiss photography software system, recorded with 2592 × 1944 pixel resolution, and saved in a 24-bit RGB JPEG file format. The lighting condition includes color cold, 0.3; color saturation, −0.2; light strength, − 0.24; and color contrast, −0.48.

For gross photography, the images of thyroids were acquired under a lighting condition using a camera (EOS 600D, Canon). The lighting condition includes shooting mode, Av/manual; focusing distance, 18.2 cm; background (for dissected thyroids), black; white balance, −2/3; and aperture, 20. To ensure the same shooting distance for each photograph of the thyroid, a coordinate paper was first focused using the lighting condition, then thyroid samples were substituted for the coordinate paper and focused again only by adjusting the shooting distance, and, finally, they were photographed.

### Quantification of Thyroid

Thyroid sizes or volumes (mm³) were determined by the sum of the areas of each section over the whole cutting region (5 to 13 serial slides, depending on the respective size of the thyroid gland), multiplied by the slice interval distance of 0.5. The thyroid area in each slide was calculated using image analysis software (Photoshop V, Adobe Photoshop Elements) as reported (2, 10).

### Alcian Blue Staining

To acquire thyroid and tracheal cartilage skeleton for thyroid gland localization, the thyroid specimens were immersed in 90% ethanol overnight after being photographed. Incubation in acetone for 6 hours was carried out before being immersed in 0.03% (w/v) Alcian blue (Cat. No 3082.3, Carl Roth, Germany) solution overnight. The staining solution was prepared in 80% ethanol and 20% glacial acetic acid. Finally, the stained samples were rinsed in 1% KOH for 4 hours and immersed in 50% glycerol for observation.

### Evans Blue Perfusion

Thyroid glands must be identified and accurately defined on the border for successful thyroid evaluation. To clarify the thyroid border in vivo for the measurement in the validation experiments, we perfused Evans blue of 0.4% (w/v, 50 µL prepared in saline) into the left ventricle of the heart. Following the perfusion, the vessel-rich thyroid became dark blue and the other around tissues light blue. The contrast sharpened the whole thyroid boundary.

### Image Processing

**Figure 3.**
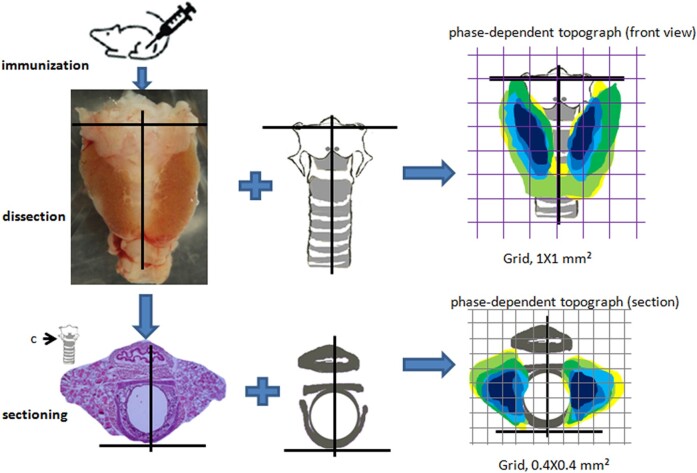
Experimental flow chart. Thyroids and their sections were collected from immunized animals. The blend of the images of thyroids and the cartilage skeleton produces phase-dependent topographs. The staining sections merged with the corresponding section landmark result in maps of site-dependent incidence in section. “c” in the inset indicates the site for sectioning.

The processing includes demarcation of the thyroid, localization of the thyroid, and a combination of images (Fig 3, Supplementary Figs S3, S12 (11)). (1) Demarcation of thyroid: Open the JPEG images with software (Photoshop V), copy and paste the images on PowerPoint with a white background. Delineate the thyroid and fill with a red color and a transparency of 70%. (2) Localization of thyroid: Draw a coordinate on the marked image as described. (3) Combination of images: Select the color and the coordinate and move on the diagram of the skeleton/coordinate. Align the coordinates so that the 2 coordinates are in correspondence with each other.

Merge all the thyroid images in the same way, leading to the production of the incidence graph of thyroid occupation in the front view and the section.

### Data Analysis

Data were presented as means ± SEM. The software SPSS (IBM SPSS Statistics for Windows, Version 11.0., IBM Corp., USA) was used for analysis of correlation, or 1-way ANOVA. A *P* < .05 was considered of statistical significance.

### Supplementary Information

Supplementary information is provided in an online data repository (11).

## Results

### Spectrum of Thyroid Volume

It is important to acquaint a spectrum of thyroid volume developed in the model at different time points so that we can determine an immunizing protocol or duration for a wide range of thyroid volume including both the minimum and maximum. With the wide range of thyroid volume (eg, large spectrum), we can better see the change in position for the alternative volume.

For this purpose, mice were immunized with the injection of either adenovirus encoding the A-subunit of the TSHR gene (Ad-TSHR) or adenovirus encoding GFP (Ad-GFP) and boosted with immunization of 3 or 4 weekly injections of the adenovirus. A detailed immunization schedule is shown in Fig 1A. At the time points—before, and at the ends of 20, 27, and 35 weeks after immunization, the mice of the indicated number (N in Fig 1B) were euthanized for thyroid volume evaluation using serial sections as reported (2, 9).

The thyroid gland in the normal adult mice was approximately 2 to 3 mm³ (Fig 1C) but was capable of enormous growth when intensely immunized with Ad-TSHR for 27 weeks, nearly achieving the summit. With immunization on, the thyroid volume increased indistinctively—by 5.8% over 27 to 35 weeks, in comparison to the increase of 78% over 20 to 27 weeks. On the contrary, in the mock immunized group (Ad-GFP), the thyroid volume could hardly change over the period (Fig 1C). Comparisons of thyroid volumes among the various time points within the Ad-TSHR group, with Tukey HST of 1-way ANOVA, resulted in a statistically significant difference (Fig 1C).

To acquire comprehensive and heterogeneous data as far as possible, a criterion that includes the minimum and maximum thyroid sizes and a large SD will be much appreciated. According to the results (Fig 1B), we selected the 35-immunized method for studying the thyroid anatomy on size and position.

### Thyroid Size and Position in Ventral View

For the study of pathological anatomy on thyroid size and position in Graves’ disease, 60 mice (Supplementary Table S1 (11)) were immunized with Ad-TSHR for 35 weeks, as indicated in Fig 1A. As a mock control group, 10 native mice were injected with saline in parallel. At the end of 35 weeks after immunization, the mice were euthanized for thyroid volume evaluation.

The thyroid size exhibited a big heterogeneity after 35 weeks of immunization. We sized the thyroids of immunization into 5 phases. The 5 phases, based on size, are 3 to 6 (T03-06), 6.1 to 9 (T06-09), 9.1 to 12 (T9-12), 12.1 to 15 (T12-15), and >15 (T > 15) in mm³. The distribution of thyroid size in the 5 phases is shown in Supplementary Fig S1 (11).

**Figure 4.**
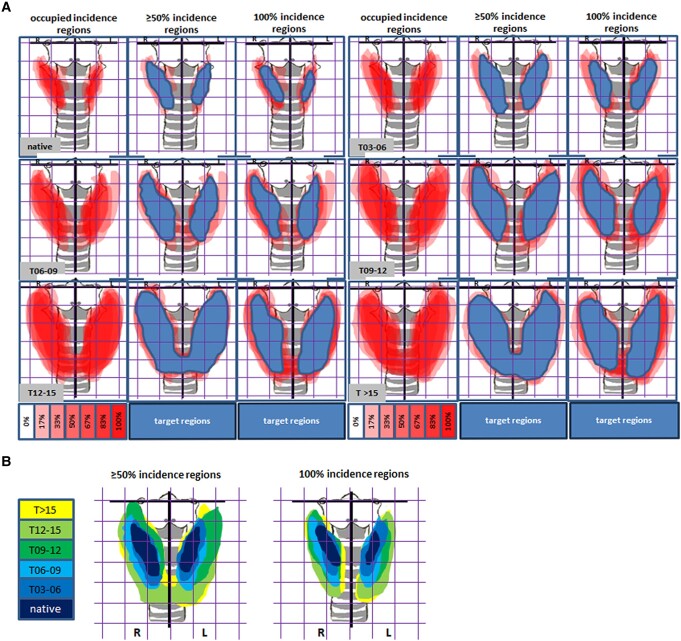
Graphs of site-dependent incidence of thyroid in front view. (A) Incidence regions during enlargement. The target regions are indicated in blue. (B) Phase-dependent topographs of ≥50% and 100% incident region in the thyroid. Grid, 1 × 1 mm²; L, left lobe; R, right lobe.

To simplify the procedure, we randomly selected 6 thyroids in each phase or group for the representatives of the native animals or the corresponding phase. For each group, we combined the 6 images of thyroids and the sketch of the skeleton in front view (Fig 3, Supplementary Fig S3 (11) with the aid of orientation of the 2-dimensional coordinates superposed on the images and the sketch. The blend resulted in the production of graphs of thyroid site-dependent incidence (Fig 4A, Supplementary Figs S4-S9 (11)).

In those graphs, we found the thyroid incidence of position was quite heterogeneous (see the column of occupied incidence regions in Fig 4). For the native group, in almost all the cases (see the column of 100% incidence regions in Fig 4A), each lobe measured approximately 2 mm long and 0.6 mm wide. It is nearly 0.5 mm from the anterior middle line in the proximal end and 1.5 mm in the distal end, centering immediately below the cricoid cartilage ring (see the column of 100% incidence regions in Fig 4A). With immunization, the thyroid underwent tremendous changes in both size and position. In the phase T > 15, each lobe approximately reached 4 mm long and 1.6 mm wide. Its central point of position moved down to 0.5 mm below the cricoid cartilage. It is nearly 0.1 mm from the anterior middle line in the proximal end and 1.5 mm in the distal end (see the 100% incidence regions column in Fig 4A).

We merged all the graphs of incidence region from the 6 phases or groups (inclusive of the native) and produced dynamic graphs during thyroid enlargement (Fig 4B, Supplementary Figs S10, S11 (11)). We observed that the enlarged thyroid mostly extended sideways and downward, and the center of gravity moved down.

### Thyroid Size and Position in 3-Dimensional View

To see thyroid volume and position alteration in depth, we collected hematoxylin and eosin-stained sections at the cutting plane (Fig 2B) 2 mm below the upper border of the thyroid cartilage (at the level of the cricoid cartilage ring). The reasons for the selection of the sections are (1) the sectioning can hit the thyroid in any case of all the phases for comparability (see the column of 100% incidence regions in Fig 4B for reference) and (2) the cricoid cartilage ring is an important landmark in the neck for location and orientation.

**Figure 5.**
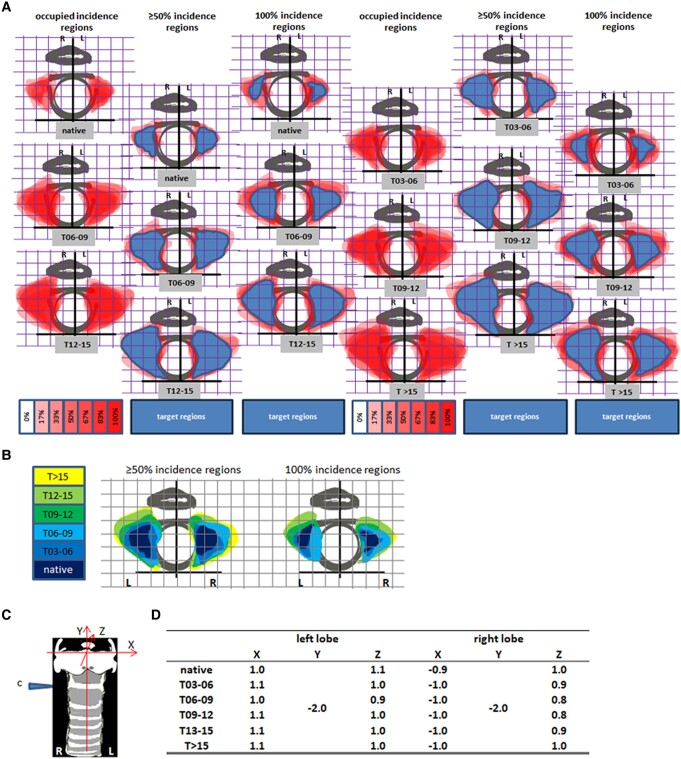
Site-dependent incidence of thyroid in section. (A) Incidence regions during enlargement. The target regions are indicated in blue. (B) Phase-dependent topographs of ≥50% and 100% incident region in the thyroid. (C) A space rectangular coordinate setting was superposed on the cartilage skeleton and used for thyroid location in the section cut at the position indicated by “c.” (D) Under the space coordinate, the 3-dimensional central points of thyroid in the column—100% incidence regions—were determined and summarized in the table with the values (in mm) of X, Y, and Z. Grid, 0.4 × 0.4 mm²; L, left lobe; R, right lobe.

Similarly, we merged 6 images of the thyroid sections with the sketch of the cricoid cartilage ring (Fig 2Ba, 3, Supplementary Fig S12 (11)) by group, with the aid of orientation of the 2-dimensional coordinates. The combination contributed to the production of graphs of thyroid site-dependent incidence in the section (Fig 5A and Supplementary Figs S13-S18 (11)).

In those graphs, we also found the thyroid incidence of the region in the section was rather heterogeneous (see the column of occupied incidence region in Fig 5A). For the native group, in most cases (see the column of ≥ 50% incidence region in Fig 5A), each lobe measured approximately 0.8 mm thick and 0.6 mm wide. It is 0.6 to 0.8 mm from the anterior middle line in the proximal end and 1.5 mm in the distal end, centering on the points (X, Y, Z) in 3-dimensional measurements (left lobe: 1.0, −2.0, 1.1; right lobe: −0.9, −2.0,1.0) (see the column of 100% incidence region in Figs 5A, C, and 5D). With immunization, the thyroid underwent tremendous changes. In the phase T > 15, each lobe reached approximately 1.6 mm thick and 1.2 mm wide. Its central point of position did hardly change. It is nearly 0.4 mm from the anterior middle line in the proximal end and 1.6 mm in the distal end (see the 100% incidence region column in Fig 5A).

We merged all the graphs of incidence region in the section from the 6 groups and resulted in dynamic graphs during thyroid enlargement (Fig 5B, Supplementary Figs S19, S20 (11)). We observed that the enlarged thyroid mostly extended anteriorly, posteriorly and laterally, and the center of gravity remained unchanged.

For the fine location of the thyroid 100% incidence, we summarized the positions of the central point of gravity with 3-dimensional values (see Fig 5D) in a table. The coordinate values in the table are the average of the minimum and maximum in the corresponding axis.

### Validation

**Figure 6.**
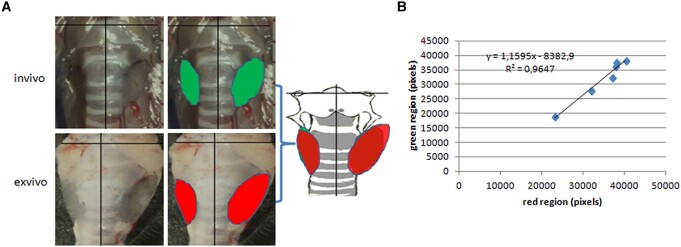
Validation was tested on a comparison between ex vivo and in vivo data. The thyroids in image (A) were labeled with different colors (in vivo - green, ex vivo - red) and combined colors. The comparison of the 2 colors (B) resulted in a statistically significant correlation. N = 6, Pearson correlation = 0.9822, *P* = .000473 (2-tailed).

There might be a discrepancy in the location of the thyroid measured between ex vivo and in vivo. The largest possibility of the discrepancy occurs in small thyroids whose borders are indistinct for some in vivo measurements. Given this reason, we selected 6 native mice (see Supplementary Table S2 (11)) for experiment validation. For validation of the method, we computationally registered the thyroid in vivo (green) and ex vivo (red) and blended the colors with the sketch of the skeleton, with the aid of the orientation of 2-dimensional coordinates (Fig 6A). A good colocalization was observed in the merged images (Fig 6A and Supplementary Fig S21 (11)), indicated by a close overlapping between the colors. To quantify the degree of the overlapping, the green, red, and overlapping areas were measured with image analysis (Photoshop V). The overall overlap rate (ratio of the blended area to the mean area of thyroid measured ex vivo and in vivo) was 86.2% (means ± SEM, 0.862 ± 0.026; N = 6), which verified the feasibility of the technique used to a rather high extent. Comparison of the areas measured in vivo and ex vivo led to a significant correlation (Fig 6B, N = 6, Pearson's *r* = 0.9822, *P* = .000437).

### Dissection of Thyroid, Cartilage Skeleton, and Esophagus

To see the surrounding structures of the thyroid, during our trial test of the experiments, we dissected some immunized mice in vivo. We observed that it was very hard to separate an intact thyroid from the trachea and thyroid cartilage. This is because the human-like lateral suspensory ligament or Berry ligament (12), a consolidation of connective tissue, connects the thyroid and the tracheal cartilages. This ligament secures each thyroid lobes to the trachea and larynx. The thyroid gland, along with the esophagus, pharynx, and trachea, is encased within a thin capsule, namely the visceral compartment of the neck, which is bound by peritracheal fascia.

## Discussion

We have established a method to induce hyperthyroidism in mice by immunization with Ad-TSHR, and the model mimics all the signs presented in the clinic (2). With the model developed, several peptides for the therapy of Graves’ disease (3-6) have been successfully tested. For investigation of the pathological anatomy of the thyroid related to the thyroid size and position, the model of long-term immunization was used. Following 35 weeks of immunization, the thyroid displayed a big heterogeneity, as naturally occurred. After the profiles on the thyroid size and position were sorted and processed, graphs of thyroid site-dependent incidence in various developing phases were produced (Figs 4 and 5, Supplementary Figs S4-S11, S13-S20 (11)). According to the different phases, the fine location of the thyroid was read out in the corresponding graph. The sonographer and radiologist need to be aware of these variants, to avoid misclassifying normal as abnormal, or vice versa.

Thyroid size is one of the important indexes for judgment of hyperthyroidism in the clinic and an indicator of T4 level and heart function. Thyroid changes in size reflect thyroid and heart function and indirect indicators of diagnosis and treatment efficiency (2). Ultrasound, computed tomography, or magnetic resonance imaging scans of the thyroid, in reference to the incidence graphs of the thyroid, would tell the degree of the thyroid enlargement.

With the advent of minimally invasive surgical techniques for treatment or diagnosis, the data on the graphs may be required for local sampling or treatment, possibly with the aid of stereotactic guidance. The graphs gave the values in X, Y, and Z for the precise location (Fig 5D). The possible stereotactic navigation allows for improved intraoperative localization that may improve the ability maximally. Thus, it may benefit fine-needle aspiration biopsy of the thyroid (13) and ultrasound-guided biopsy technique (13) for fine localization by increasing confidence, reducing surgical stress, and leading to greater success in sampling.

We integrated all the graphs of site-dependent incidence of thyroid from the different phases into a dynamic phase-dependent topography (Figs 4B, 5B). From the topography, we found the thyroid changes in position and volume during enlargement. The tremendous changes in the thyroid's position and size may cause clinical symptoms and signs. The rapid expansion of the thyroid down- or backward may cause dysphagia or shortness of breath as a result of the thyroid gland directly compressing the swallowing and breath organs. The rapid expansion of the thyroid sideways or frontward may display the sign of goiter.

The graphs and the table (Fig 5BD) might also assist in local therapy in animal research. For the transition from the findings to the clinic, future studies should focus on developing strategies to identify the relationship between mouse and human grave disease in thyroid position and size, by noninvasive imaging techniques. The studies will help identify any patterns or trends that may be associated with the development of the disease in humans and find new and effective treatment strategies for Graves' disease.

For an accurate interpretation of the results, validation of the protocol should be noted. For the validation experiments, we perfused the animals intracardially with Evans Blue when the thyroid gland in vivo was unclear in the border (Supplementary Fig S21 (11)). The normal gland, in some cases, especially in the small size, is usually unclear on inspection, and it is often difficult to define the border. On the contrary, the thyroid ex vivo is very conspicuous under the black background. Therefore, the data on thyroid on the position and size were mostly collected ex vivo. The data on the thyroid position and volume collected ex vivo is not different from that in vivo, as reflected by the outcome of validation tests. It is not surprising that the thyroid volume and location do not change in measurement after dissection. Like human surrounding structures of the thyroid (12), the ligament and fascia fix the gland and ensure that the position and shape of the thyroid remain unchangeable after the dissection, in reference to the cartilage skeleton.

It would be of great value if the data were collected from autopsies on humans. Given the rarity of the dead at different phases of thyroid enlargement, such a trial is unfeasible. However, we collected the data from mice because the great similarities in multiple aspects of the thyroid gland between humans and small rodents have facilitated the rapid translation of experimental findings to the clinical realm (14). Knowledge of thyroid changes in size and position of the model might be helpful for both basal and clinical studies.

## Data Availability

All data generated or analyzed during this study are included in this article, or the data repositories (11) listed in the References.
